# Effect of Long-Term 3D Spheroid Culture on WJ-MSC

**DOI:** 10.3390/cells10040719

**Published:** 2021-03-24

**Authors:** Agnieszka Kaminska, Aleksandra Wedzinska, Marta Kot, Anna Sarnowska

**Affiliations:** 1 Mossakowski Medical Research Centre, Translational Platform for Regenerative Medicine, Polish Academy of Science, 02-106 Warsaw, Poland; akaminska@imdik.pan.pl (A.K.); awedzinska@imdik.pan.pl (A.W.); 2 Mossakowski Medical Research Centre, Department of Stem Cell Bioengineering, Polish Academy of Sciences, 02-106 Warsaw, Poland; mkot@imdik.pan.pl

**Keywords:** mesenchymal stem cells, mesenchymal stromal cells, 3D culture, neurospheres, spheroids, pluripotency, neural, quiescence

## Abstract

The aim of our work was to develop a protocol enabling a derivation of mesenchymal stem/stromal cell (MSC) subpopulation with increased expression of pluripotent and neural genes. For this purpose we used a 3D spheroid culture system optimal for neural stem cells propagation. Although 2D culture conditions are typical and characteristic for MSC, under special treatment these cells can be cultured for a short time in 3D conditions. We examined the effects of prolonged 3D spheroid culture on MSC in hope to select cells with primitive features. Wharton Jelly derived MSC (WJ-MSC) were cultured in 3D neurosphere induction medium for about 20 days in vitro. Then, cells were transported to 2D conditions and confront to the initial population and population constantly cultured in 2D. 3D spheroids culture of WJ-MSC resulted in increased senescence, decreased stemness and proliferation. However long-termed 3D spheroid culture allowed for selection of cells exhibiting increased expression of early neural and SSEA4 markers what might indicate the survival of cell subpopulation with unique features.

## 1. Introduction

Mesenchymal stromal/stem cells (MSC) were discovered by Friedenstein in 1966 [1] and since that time most of the researchers have used 2D culture condition to expand this population. The adherence is listed as one of the criteria to revise cells as MSC [2]. Monolayer culture system allows MSC to attach to the surface just like in natural environment and to expand in two dimensions. In spite of its widespread, this method has multiple limitations, and it is discussed how close is to the natural cell environment of MSC [3,4].

Currently, cell cultures are cultivated more and more often in 3D conditions as an alternative for 2D conditions. Spheroid culture, one from the multiple solutions, provides cell-to-cell contacts and intercellular signaling what resembles the environment of tissue [5]. Moreover, such a method of culture is also supposed to imitate the natural cell niche with the stem cells preserved in it.

Spheroid is the floating aggregate of cells with visible changes across its structure. The core consists of proliferating cells, whereas cells from external layer might differentiate and migrate. In order to mimic the natural conditions, MSC has also started to be cultured as 3D aggregates. MSC spheres are described to be formed by using different protocols including low attachment surface [6,7], hanging drop culture [8,9], scaffolds [10], and even the bioreactors [11]. Few research groups tested also neurosphere assay, proposed for neural stem cells (NSC) [12,13] to achieve MSC spheroids. Culture media for neurospheres contain epithelial growth factors (EGF) and basal fibroblast growth factor (bFGF) but no serum.

Most of the experiments conducted with MSC-neurospheres were focused on acquisition of neural phenotype. it was suggested that 3D culture condition could improve neural differentiation of MSC. In spite of improvement of neural differentiation under 3D conditions, evidences of receiving fully functional neuronal cells from MSC populations are limited. Still there is a demand for efficient protocol, which could be used in clinic. Except analysis of neural phenotype, other aspects such as proliferation, senescence and stemness were not so broadly taken into consideration during research. Moreover, majority of MSC spheroids experiments were a short-term—3D cultured did not last up to seven days in vitro of culture (div) [4] and results were obtained usually during first three div. There were little evidences whether observed effects were transient or constant whether how cells would react for prolonged 3D conditions. Even less is known about the influence of 3D condition on stem cell niche: effect on surface markers expression, commitment of specific type of MSC or interaction between cells. That would explain why some cells survive in 3D conditions and how we could select with better properties.

In the present study, Wharton Jelly derived (WJ)-MSC were cultured in two different culture conditions as the standard, monolayer 2D culture or as 3D culture. Both cultures were conducted parallel for about 20 div—the time required to achieve three passages during standard 2D MSC culture. Cells derived from spheroid culture were compared to those cultured as monolayer regarding such properties as cell senescence, rate of proliferation, capability to form the colonies, and pluripotent and neural gene expression.

## 2. Materials and Methods

### 2.1. WJ-MSC Isolation and Primary Culture

Human umbilical cords were acquired from full-term deliveries with the written consent of mother according to the Ethics Committee of Warsaw Medical University guideline (KB/213/2016). Cords (15–20 cm) transported in phosphate buffer saline (PBS) solution (PBS; Sigma-Aldrich, Saint Louis, MO, USA) with mix of Penicillin-Streptomycin-Amphotericin B (1:100, Gibco, Thermo Fisher Scientific, Waltham, MA, USA) were cut with lancet to 2–3 mm in thickness slices. The cylindrical fragments of Wharton Jelly (WJ) of 2–3 mm diameter were obtained from the slices of umbilical cord using the diameter biopsy punch (Miltex, GmbH, Viernheim, Germany). Explants were transferred to six well cultured plates and culture in the standard cell culture medium for WJ-MSC: DMEM (Gibco), 10% human platelet cell lysate (Macopharma, Tourcoing, France), mix of penicillin, streptomycin amphotericin B (1:100; Gibco, Thermo Fisher Scientific), 2 µg/mL heparin (Sigma-Aldrich). Conditions for cell culture were following adherent surface, 37 °C temperature, 95% of humidity, 5% concentration of CO_2_, and 5% concentration of O_2_. The culture medium was replaced every 2 days for 14 div. When the cells migrated out of the explant and the culture reached semiconfluence, the cells were detached with Accutase Cell Detachment Solution (Beckton Dickinson, Franklin Lakes, NJ, USA) and counted.

WJ-MSC were cultured in conditions described above until the end of 3 passage (initial population of WJ-MSC). After the 3rd passage, cells were collected and divided into two group—part of them was cultured as a spheroids (3D cultured WJ-MSC) and the rest were continually cultured in previous cell culture conditions until the 7th passage (2D cultured WJ-MSC).

### 2.2. Spheroid Culture

WJ-MSC from the 3rd passage were collected and seeded on anti-adhesive 6 well plates (Nunclon Sphera, Thermo Fischer Scientific, Waltham, MA, USA) at the high density of 30 × 10^3^/cm^2^ in 5% O_2_. Cells were cultured in medium DMEM/F12 (Gibco) containing mix of penicillin, streptomycin, amphotericin B (1:100) (Gibco), L-Glutamine 200 mM (1:100) (Gibco), N2 supplement (1:100; Biotechne, Minneapolis, MN, USA) and EGF (20 ng/mL; PeproTech, London, UK). In 3 div, Medium was replaced, and new medium was started to use from this point of culture—Neurobasal Medium (Gibco, Thermo Fischer Scientific) with mix of penicillin, streptomycin, amphotericin B (1:100; Gibco, Thermo Fischer Scientific), L-Glutamine 200 mM (1:100; Gibco, Thermo Fischer Scientific), B27 supplement (1:50; Gibco, Thermo Fischer Scientific), EGF (20 ng/mL; PeproTech) and bFGF (20 ng/mL; PeproTech).

WJ-MSC spheres were cultured in parallel to standard 2D culture—until the standard culture acquire the 7th passage (about 20 div of culture). After that time spheroids were dissociated to obtain single cells with Accutase Cell Detachment Solution (Becton Dickinson) and seeded again to the 2D culture in standard medium. Reseeded cells were used to measure colony forming unit frequency (CFU-F), population doubling time (PDT), senescence processes, gene expression level, and to perform immunocytochemistry staining.

### 2.3. Flow Cytometry Analysis

Cells were detached with Accutase Cell Detachment Solution (Beckton Dickinson) and washed in PBS. Required cell number (1 × 10^6^) was resuspend in cold Stain Buffer (Beckton Dickinson) and used for further flow cytometry analysis. Cell markers were analyzed with Human MSC Analysis Kit (Beckton Dickinson) containing antibodies conjugated with fluorochrome against following antigens: CD73, CD90, CD105 (positive markers), CD11b, CD19, CD34, CD45, and PE (negative markers) (Table 1). Cells were incubated in diluted antibodies in the dark for 30 min. After incubation, cells were washed twice with Stain Buffer (Beckton Dickinson) and resuspend in Stain Buffer. Resuspended cells were analyzed using FACS Canto II (Beckton Dickinson) with FACSDiva Software (Beckton Dickinson) and FlowJo 10 (Beckton Dickinson).

In 3rd and 10th div of 3D culture, spheroids were dissociated and resuspended in Stain Buffer. Before running the sample, cells were filtered through 30 µm filter (Miltenyi Biotec, Bergisch Gladbach, Germany) to avoid dublets. Cells were analyzed to compare the change in size with flow cytometry FACS Canto II (Beckton Dickinson) with FACSDiva Software (Beckton Dickinson) and FlowJo 10 (Beckton Dickinson).

### 2.4. Live-Dead Staining

Aggregates were stained with mix of ethidium homodimer-1 (8 µM, EthD-1) and Calcein AM (Cal-AM) (0.1 µM, Invitrogen, Thermo Fischer Scientific, Waltham, MA, USA) to confirm the viability of cells in spheroids. Spheroids or single cells derived from spheroids were incubated with staining mixture for 45 min in room temperature in a darkness. Stained cells were observed in fluorescence microscope Axio Vert.A1 (Carl Zeiss, Oberkochen, Germany).

### 2.5. CFU Assay

WJ-MSC from initial population, 2D culture and 3D culture were seeded on 6-well plate in the amount of 100 cells per well. Cells were cultured for 10 div in standard conditions. Then, cells were washed with PBS, fixed with 4% PFA for 15 min and again washed with PBS. Fixed cells were stained with 0.5% toluidine blue for 20 min and washed with distilled water after staining. The number of colonies containing 50 cells or more were counted, and CFU-F was calculated as a percentage of seeded cells.

### 2.6. Senescence Assay

The cells senescence was analyzed with Senescence Cells Histochemical Staining Kit (Sigma-Aldrich) in initial population, 2D culture, and 3D culture of WJ-MSC. Cells from 2D cultures grew until the confluence reached 50–60% while the cells from dissociated spheroids were cultured 48 h in standard conditions and then the assay was performed.

Cells were washed with 1×PBS and then fixed with Fixation Buffer (provided with kit) for 6–7 min in room temperature. Then, cells were washed 3 times with 1×PBS. After washing, cells were incubated overnight in 37 °C with Staining Solution (prepared according to the protocol). Next day, total cell number was count as well as blue-stained cell number. The percentage of β-galactosidase positive cells was calculated.

### 2.7. Proliferation Analysis

The cell proliferation was analyzed in the initial population, 2D culture, and 3D culture. WJ-MSC were seeded at a density 2000 cells/cm^2^ and cultured in standard conditions until the 80% confluence was reached. Then cells were collected, counted, and re-seeded again at initial density. PDT and cumulative population doublings (cPD) were calculated for the next 4 passages, based on total cell number at each passage. The PDT value was calculated with the following formula:

PDT = ((t − t_0_) × log2)/(logN − logN_0_)

where N is the number of cells obtained at the end of the passage, N_0_ is the initial number of seeded cells, and t − t_0_ is the duration of passage (counted in days).

### 2.8. Cryostat Sectioning

Spheroids were collected, fixed in 4% PFA for 15 min and washed twice with PBS. Then, PBS was replaced with 7.5% sucrose solution and incubated overnight. Next day, solution was replaced with 15% sucrose solution and 30% sucrose solution. Then, spheroids were embedded in medium for frozen tissue specimen (OCT Sakura Tissue-Tek, Sakura Finetek Europe, Alphen aan den Rijn, The Netherlands) and moved to −80 °C. Spheroids were cut with cryostat for the 20–30 µm thickness sections. Sections were collected on APTEX (3-Aminopropyl) triethoxysilane) coated glass microscope slides, stored in −20 °C and used for immunocytochemical staining.

### 2.9. Immunocytochemistry

Immunocytochemistry was performed to detect pluripotency and early neural markers in initial population, 2D culture and 3D culture of WJ-MSC. WJ-MSC were washed with PBS and fixed in 4% PFA for 15 min. Samples were permeabilized with 0.2% Triton X-100 (Sigma-Aldrich) for 15 min and then washed with PBS. After incubation with 10% Goat Serum (Sigma-Aldrich) for 1 h primary antibodies were applied for 24 h in 4 °C (Table 2). Next day, cells were washed with PBS and then incubated with the secondary antibodies conjugated with fluorochrome for 1 h (Supplementary Table S1, Supplementary Figure S1). Cell nuclei were stained with Hoechst 33342 dye (1 µg/mL; Sigma-Aldrich). The analysis was performed using confocal microscope Zeiss LSM780 (Carl Zeiss).

### 2.10. Real Time-Quantitative Polymerase Chain Reaction (RT-qPCR)

Total RNA was isolated from initial, 2D populations, and 3D cultured population using the following kits: Total RNA Mini Plus kit (A&A Biotechnology, Gdynia, Poland) and Total RNA Mini Plus Concentrator (A&A Biotechnology) according to the manufacturer’s protocols.

RNA was eluted with 20 µL of RNase-free H_2_O (Sigma Aldrich). The quantity and the quality of RNA were assessed using a NanoDrop 2000 spectrophotometer (Thermo Scientific). The elimination of genomic DNA (gDNA) contamination in all RNA samples was performed using a Clean up RNA Concentrator (A&A Biotechnology).

RNA samples were stored at −80 °C until were further used. A complementary strand of DNA (cDNA) from RNA was generated using a High-Capacity RNA-to-cDNA™ Kit (Applied Biosystems, Thermo Fischer Scientific, Waltham, MA, USA) according to the manufacturer’s instructions. Following the reverse transcription, samples were diluted in RNase-free water and stored at −20 °C until subsequent testing.

Quantitative polymerase chain reactions were performed using SYBR green Master Mix (Applied Biosystems) and specific primers (Table 3) with the 7500 Real Time PCR System (Applied Biosystems). The relative amount of RNA was calculated with the comparative delta-delta Ct method (2^−ΔΔCt^) and gene expression was normalized using β-actin (ACTB). Gene expression was compared with the mean level of the corresponding gene expression in cells from initial population (3rd passage of WJ-MSC culture) and expressed as n-fold ratio.

### 2.11. Statistics

Two-group comparisons were performed with Student’s test, whereas multiple groups used one-way analysis of variance (ANOVA). The results are presented as mean values of 3 independent experiments ± SD (* < 0.05, ** < 0.01, *** < 0.001, **** < 0.0001), each experiment was performed with cells obtained from one donor. Statistical analysis was conducted with GraphPad Prism v. 7.00 software.

## 3. Results

### 3.1. Characteristics of WJ-MSC Cultured in 2D and 3D Conditions

**Figure 1:**
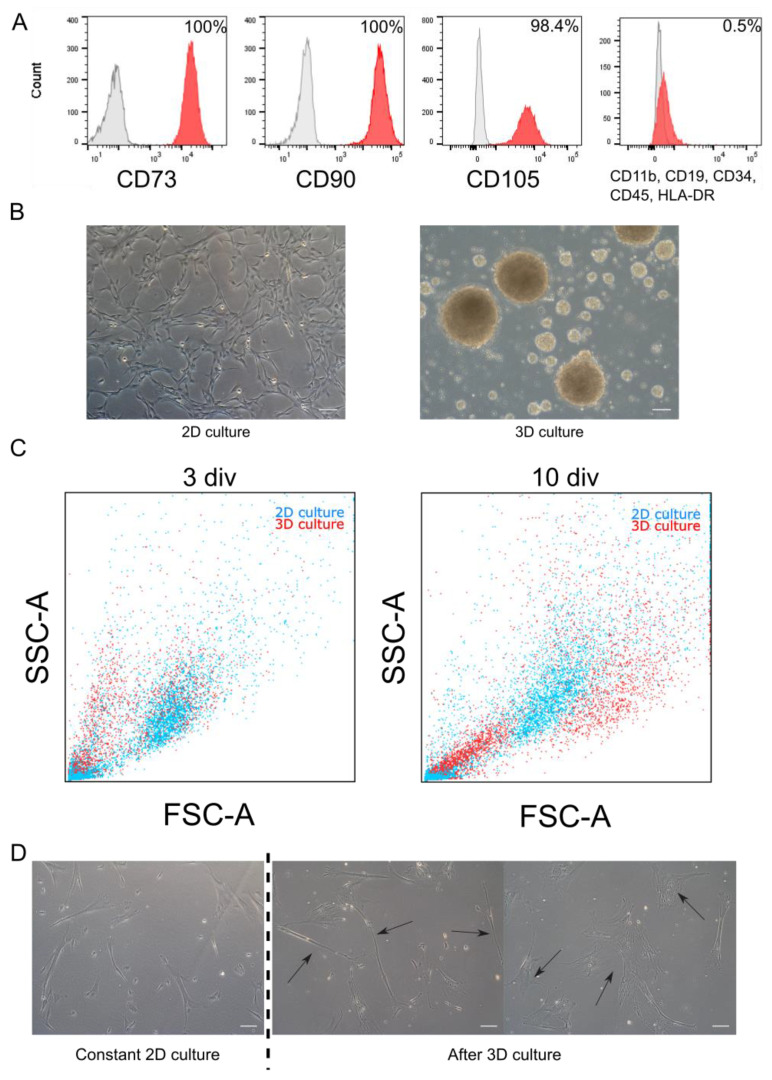
Morphology and phenotype of WJ-MSC cultured in monolayer and as spheroids. (**A**). Flow cytometry analysis. Initial population of WJ-MSC used to experiments expressed specific MSC markers (CD73, CD90, and CD105) and less than 1% of WJ-MSC expressed negative markers (CD11b, CD19, CD34, CD45, and HLA-DR). Red histogram—analyzed marker, grey histogram—isotype control. (**B**). Morphology of 2D and 3D cultured WJ-MSC—monolayer culture of WJ-MSC from 4th passage (up) and WJ-MSC 4 div after formed spheroids (down). (**C**). Flow cytometry—Forward scatter (FSC) and Side scatter (SSC) analysis for 3D culture (spheroids) and 2D (monolayer). Three days in vitro (div) and 10 div after sphere induction, there were differences in size of cells between 2D and 3D culture. (**D**). Different morphology of cells cultured as spheroids after reseeding into 2D conditions. Right: WJ-MSC cultured constantly in 2D for 8 passages. Center and left: WJ-MSC from 3D reseeded to 2D condition—visible two different types of morphology: narrow and small (center) and flat and broad (left) cells. White scalebars represent 100 µm.

WJ-MSC chosen for experiments exhibited characteristic features of MSC such as morphology and expression of markers. Cells were adherent to the surface and presented spindle, fibroblast-like morphology. Flow cytometry analysis revealed that cells expressed specific mesenchymal markers (CD73, CD90, and CD105). Less than 1% of WJ-MSC expressed negative markers for MSC (CD34, CD11b, CD19, CD45, and HLA-DR) (Figure 1A). Above described features are accordant with the minimal criteria for MSC established by The International Society for Cellular Therapy.

WJ-MSC were cultured until the third passage in the monolayer with standardly used culture medium. Then, collected cells were divided into two groups: first group was cultured constantly as monolayer (2D cultured), while the second—as spheroids (3D culture) (Figure 1B). Both cultures were conducted for time required for three passages (about 20 div). Then spheroids were dissociated and seeded again to 2D conditions—to observe changes of WJ-MSC. In further analysis, following populations were compared: initial population—third passage of standard WJ-MSC culture; 2D culture—seventh passage of standard WJ-MSC culture; and 3D culture—WJ-MSC cultured as spheroids for 20 div and then reseeded to standard conditions.

Applied 3D method, slightly modified in our laboratory succeeded in sphere formation by WJ-MSC. WJ-MSC were cultured on anti-adhesive surface with the presence of supplements and mitogens (EGF and bFGF)—similarly to the neurospheres formed by NSC. Diameter of WJ-MSC spheroids varied from 20 µm to even 500 µm; however, average size oscillated between 40 and 120 µm. Spheres were cultured up to 20 div; however, the best morphology was observed in the first five div of 3D culture—in later stages spheres spontaneously disintegrated.

Flow cytometry analysis revealed the change in size of single cells—differences were noticed during the cultivation time, as well as between 2D and 3D culture models (Figure 1C). In young spheroids (three div) small, round cells predominated, compared to parallelly cultured 2D cells, whereas in old spheroids (10 div) we could distinguish an increase in the number of large cells. During 2D culture, the ratio of both cells subpopulations (small and large) did not change remarkably.

Adherent properties in WJ-MSC were still detectable after long-term 3D. Although cell morphology was similar to those acquired during standard culture, some differences occurred more frequently (Figure 1D). After 3D culture, we distinguished three subpopulation of cells—standard fibroblast-like cells similar to standard 2D culture; narrow and spindle cells with improved neural potential; and very broad cells indicating senescent morphology.

**Figure 2:**
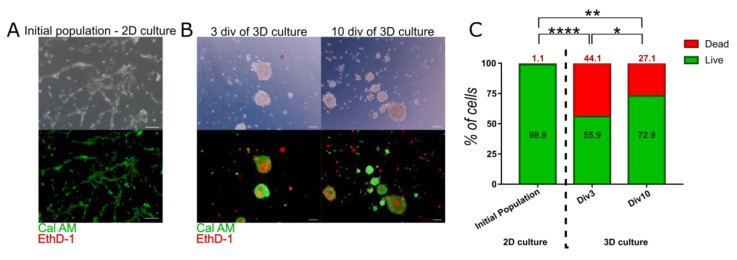
Viability of 3D cultured WJ-MSC—Calcein AM (Cal AM) and Ethidium homodimer-1 (EthD-1) staining. Cal AM stains live cells in green, while EthD-1 stains dead cells in red. Double stained cells are early apoptotic cells. (**A**) Initial population of WJ-MSC contained live cells with almost no dead cells. Scale bars: 100 µm. (**B**) Viability of cells in spheroids—after 3 and 10 div (day in vitro) of 3D culture. Observed darker shade in the contrast phase corresponds to dead cells visible. Scale bars: 100 µm. (**C**) Analysis of live, dead, and early apoptotic cells in initial population and 3D cultured population for 3rd and 10th (div). The results are presented as mean values of 3 experiments ± SD, for * < 0.05, ** <0.01, **** < 0.0001.

Live-dead staining using EthD-1 and Cal AM revealed that most of the cells cultured in monolayer were alive (98.91% ± 0.26) (Figure 2A), whereas spheroids contained significantly more dead cells inside (Figure 2B,C). Number of alive cells was reduced to 55.9% ± 6.18 and 72.87% ± 5.29 in 3 and 10 div respectively (Figure 2C). The increase in viability between early and late stage of spheroid culture was also significant.

### 3.2. Physiological Properties of WJ-MSC Cultured in 3D Conditions

Spheroids culture conditions change not only the morphology of WJ-MSC, but also influence on the physiological features of the cells. We compared culture features such as: doubling ratio of cells, induction of senescence process and content of stem fraction in population. For this purpose, WJ-MSC spheroids were dissociated to the single cells after 20 div of culture and reseeded to monolayer culture conditions. Results of assays obtained from 3D cultured WJ-MSC were compared to those observed in the initial population of WJ-MSC (from passage 3) and WJ-MSC continuously cultured in 2D conditions (from passage 7).

**Figure 3:**
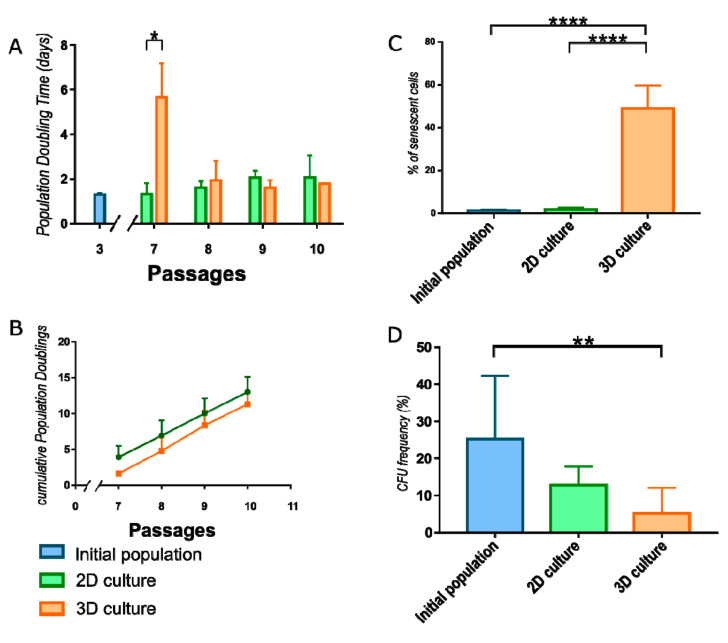
Characteristic of WJ-MSC cultured in different conditions. (**A**) Population doubling time (PDT) for WJ-MSC cultured in 2D and 3D. 3D cultured WJ-MSC divided more slowly than 2D cultured WJ-MSC during the first passage. However, there are observed no differences in next 3 passages. (**B**) Cumulative Population Doublings calculation revealed shift between 2D and 3D cultured populations. (**C**) Senescence process analysis. Number of cells expressing β-galactosidase was significantly higher in 3D cultured WJ-MSC than in initial and 2D cultured populations. (**D**) Colony forming unit (CFU) frequency reduced significantly during 3D culture of WJ-MSC. Following populations of WJ-MSC were used: from 3rd passage (initial populations), from 7th passage (2D culture) and cultured as spheroids (3D culture). The results are presented as mean values of 3 experiments ± SD. For * < 0.05, ** <0.01, **** < 0.0001.

WJ-MSC were monitored after reseeding to monolayer conditions to calculate Population Doubling Time (PDT) required for each of next four passages. During first passage after 3D cultured, cell proliferation decreased compared to cells constantly cultured in monolayer (Figure 3A). In further passages, 3D cultured WJ-MSC restored the doubling rate and divided in the same ratio as 2D cultured WJ-MSC (Supplementary Table S2). There is a shift between passages of 2D and 3D cultured populations when cumulative value is analyzed (Figure 3B).

To evaluate the effect of the culture method on cell senescence, the β-galactosidase activity was measured. Spheroid-cultured WJ-MSC exhibited significantly higher activity of β-galactosidase (49.04 ± 10.72) than initial population of WJ-MSC (1.24 ± 0.59) and WJ-MSC continuously cultured as monolayer (1.84 ± 0.93) (Figure 3C).

ISCT recommends the Colony forming unit frequency assay (CFU-F) to confirm the stemness of MSC and estimate the fraction of stem/progenitor cells in the population. The values of CFU-F from initial population of WJ-MSC (19.63± 9.5) and 2D cultured WJ-MSC (12.78 ± 5.02) does not differ significantly. However, 3D cultured WJ-MSC indicated significantly reduced CFU-F than initial population (5.11 ± 6.95) (Figure 3D).

### 3.3. Neural and Pluripotent Markers Expression in 3D Cultured WJ-MSC

**Figure 4:**
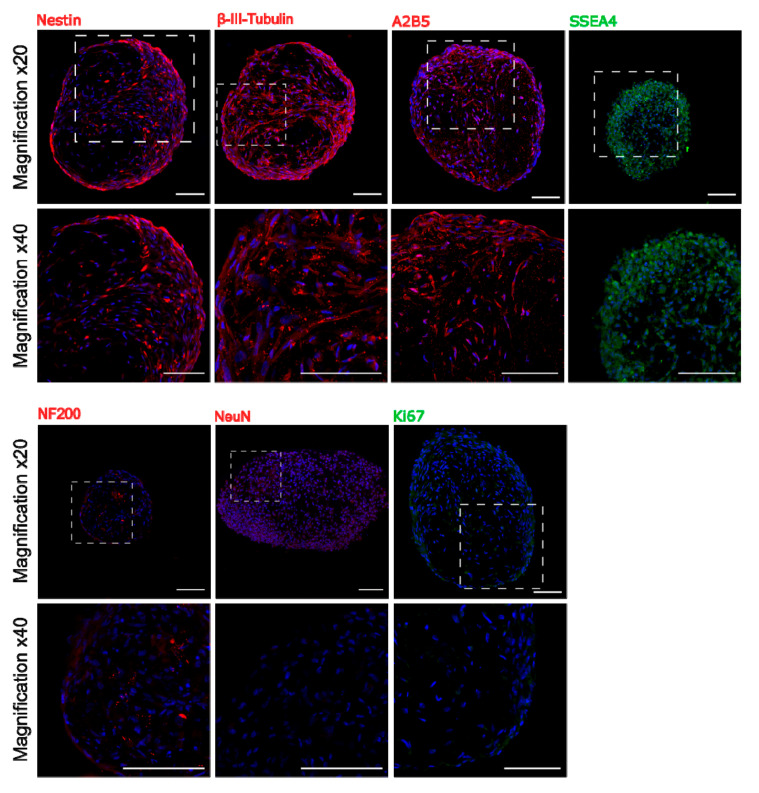
Immunocytochemistry staining of neural and pluripotent markers in sectioned WJ-MSC spheroids in two different magnifications. Early neural markers are also expressed inside the sphere, whereas more mature neural markers were not detected. Spheroids were collected between 10 and 20 day in vitro of 3D culture. Scale bars: 100 µm.

To find out whether 3D culture condition predisposes to increase expression of early neural as well as pluripotent markers, immunocytochemical analysis was performed. Staining of sectioned spheroids revealed the presence in the sphere the early neural markers: Nestin and β-III-Tubulin, early oligodendrocyte marker A2B5 as well as pluripotent surface marker SSEA4. However, we did not observe more mature markers as NF200 or NeuN inside the sphere (Figure 4). Double staining for SSEA4 and Caspase-3 showed that most of SSEA-4 positive cells remained alive (Supplementary Figure S2).

**Figure 5:**
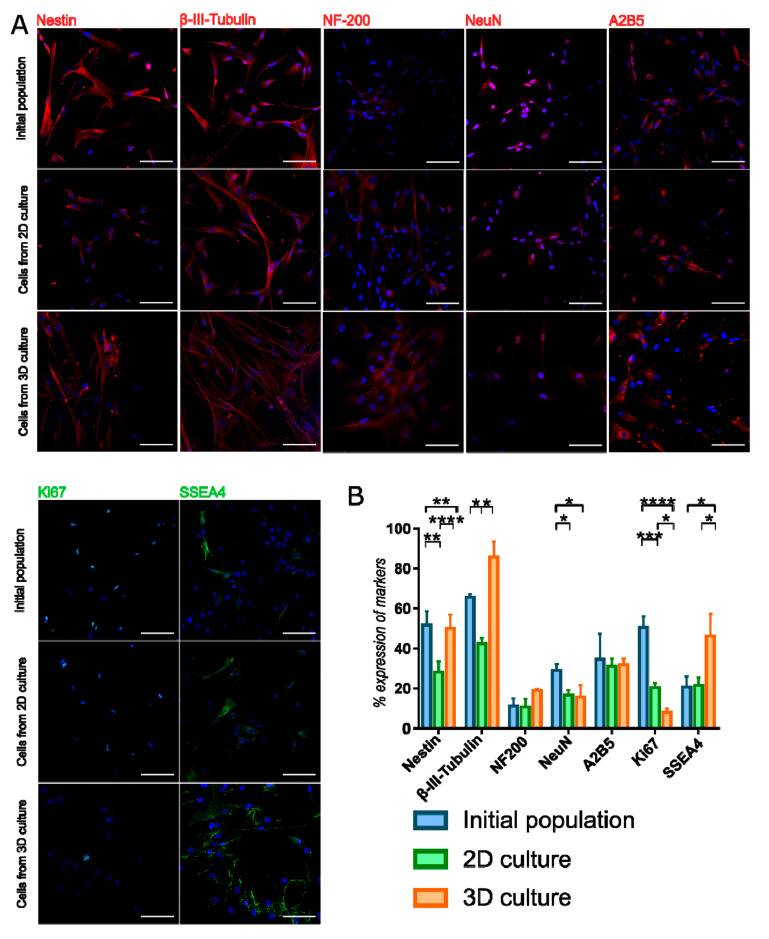
Immunocytochemistry staining of neural and pluripotent markers in WJ-MSC cultured in different conditions. (**A**) 3D cultures changes the expression of some neural and pluripotent markers what is observed even after reseeding to 2D culture. Scale bars: 100 µm. (**B**) Analysis of marker expression in WJ-MSC populations. Following populations of WJ-MSC were used: cells from 3rd passage (initial populations), cells from 7th passage (2D culture) and cells cultured as spheroids after reseeding to 2D (3D culture). The results are presented as mean values of 3 experiments ± SD. For * < 0.05, ** <0.01, *** < 0.001, **** < 0.0001.

3D population seeded again on the monolayer also displayed early neural and oligodendrocyte markers—the expression was compared to initial population and 2D constantly cultured WJ-MSC (Figure 5, Supplementary Table S3). Expression of Ki67—proliferation marker—was decreased in 3D cultured WJ-MSC compared to initial and 2D cultures, what confirms previously described PDT and CFU-F observations. Early neural markers—Nestin and β-III-Tubulin—were presented more frequently in 3D cultured WJ-MSC than in 2D cultured WJ-MSC. β-III-Tubulin expression in 3D WJ-MSC was even higher than in initial population. However, 3D cell culture conditions did not increase the expression of more mature markers NF-200 and NeuN. Changed culture condition did not affect the expression of A2B5. Expression of SSEA4—pluripotent surface glycosphingolipid—was increased in 3D cultured WJ-MSC, what may indicate that SSEA4-positive cells more favorably survives during long-termed 3D culture.

**Figure 6:**
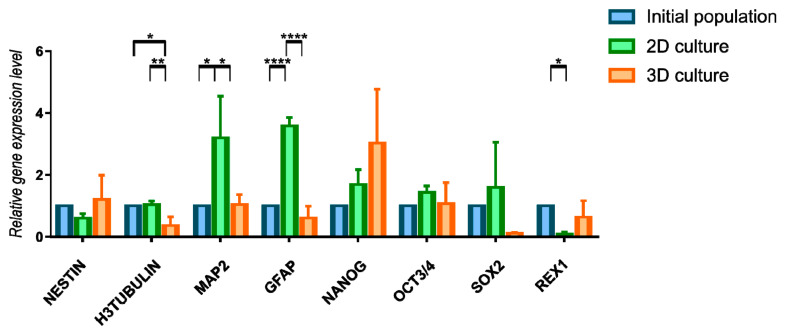
Relative gene expression level (fold change, mean ± SD) of neural and pluripotency phenotype in 2D and 3D WJ-MSC cultures. Quantitation of these genes was determined relative to ACTB as a housekeeping gene by quantitative real-time PCR. Changes in gene expression in WJ-MSC cells cultured in 2D and 3D conditions are shown relative to that in cells grown in the initial population. Following populations of WJ-MSC were used: from 3rd passage (initial populations), from 7th passage (2D culture), cultured as spheroids and transferred to 2D (3D culture). Results shown are the mean of 3 independent RNA isolations, for * < 0.05, ** <0.01, **** < 0.0001.

3D culture influence was also assessed by the expression changes in genes characteristic for neural or pluripotent phenotype (Figure 6, Supplementary Table S4). Due to the huge fluctuations between genetic material from different isolations we did not reported significant changes upon a long-term 3D cells culture when it comes to early neural marker Nestin and pluripotency genes Nanog, Sox2, Oct3/4, and Rex1. Tendency of increased expression of Nestin and Nanog genes may indicate the maintenance of early undifferentiated state of cells during 3D cell culture. Other analyzed markers—H3Tubulin (early neural marker), MAP2 (mature neural marker) and GFAP (astroglial marker) revealed some changes between initial population and two methods of further culturing. 2D conditions was more favorable for expression of MAP2 and GFAP. Results obtained from gene expression and immunocytochemistry analysis are not fully consistence, however it may be connected with differences between transcriptional and protein level of analyzed markers. We also performed gene expression analysis for RNA isolated directly from 3D spheroids—without reseeding to 2D (Figure S3). We observed the changes between two timepoints of analysis—Nestin was expressed on higher level in 3D spheroids than 48 h after reseeding to 2D culture. We observed this tendency also for other genes; however, differences were not significantly important.

## 4. Discussion

Umbilical cord contains Wharton Jelly, which is commonly used source of MSC in stem cell research [14,15]. Different factors and culture conditions are proposed to improve either stemness properties or differentiation potential of WJ-MSC. Our group is especially interested in establishment of protocol inducing differentiation of WJ-MSC toward neural lineage—due to limited alternatives for regeneration of neural system. Although neural differentiation of MSC is controversial, due to the lack functional evidence in vivo, our group confirmed the presence of voltage-sensitive and ligand-gated channels in differentiating neural stem-like cells derived from the nonhematopoietic fraction of human umbilical cord blood [16]. As MSC populations is highly heterogenous [17], we assume initial selection of pro-neural or undifferentiated cells would improve the effect of further neural differentiation. We continued in targeting our research on WJ-MSC and looked for the manner of increasing their neural/undifferentiated potential as we had reported in WJ-MSC spontaneous expression of neuroglial markers such as β-III-Tubulin, GFAP, NF-200 probably due to their premature origin [18]. Change of culture spatial structure appeared to be promising factor that we would like to confront.

The present studies were focused on the effect of prolonged 3D culture system of MSC. We tried to find out if applied culture conditions would result in obtaining population with better proliferation potential, younger phenotype or increased clonogenic abilities like in the cell niche. What is more, we examined the influence on gene expression and presence of markers responsible for neural or pluripotent phenotype—which direction cells would follow after 3D culture. WJ-MSC were cultured as spheroids in long termed culture for about 20 div to examine physiological changes. We compared features of 3D cultured cells with initial population derived from early passage (third passage) as well as population cultured constantly in monolayer up to the seventh passage. Clonogenicity, proliferation potential, senescence, and expression of neural and pluripotency markers were analyzed to confirm whether long-term 3D conditions are favorable for MSC.

We confirmed that despite their adherent properties, WJ-MSC can be directed to form the spheres in culture medium dedicated for NSC formed neurospheres. Spheroids can be generated also from other tissues containing MSC such as bone marrow [19], adipose tissue [20], or dental pulp [21] as well as other [21,22,23,24]. Main purpose of 3D neurosphere culture is to selectively isolate and culture the subpopulation of cells with higher neural differentiation capacities.

WJ-MSC in spheroids still exhibited strong adhesive properties—even undissociated spheres in standard culture medium settled down and attached to surface. 3D culture also did not change expression of typical MSC markers (CD73, CD90, and CD105) and multipotent differentiation into mesodermal lines [25,26]. Some research groups observed even enhanced mesodermal differentiation after 3D culture [7,26]. Spheroid culture led to morphology changes, visible especially during the first passage after reseeding on 2D [6,8,11,27]. Except WJ-MSC with characteristic fibroblast-like morphology, we observed more small cells with round or spindle shape. Acquisition of round shape and then elongation is typical for neural cell maturation from progenitors to immature neurons [28]. Morphological changes were also observed in non-neural cells undergoing neuronal differentiation [29]. Small size of cells after 3D culture could be also the effect of cell reorganization in spheroids and decreased packing density [11,27]. Unfortunately, we also noticed more large, flat cells connected with the loss of stemness state and higher senescence process [30,31]. We speculate that 3D culture could promote the survival of quiescent cell subpopulation.

3D cultured WJ-MSC significantly differed in cell division ratio than initial WJ-MSC population and 2D cultured WJ-MSC. What is more, immunochemistry staining for Ki67—proliferation marker—confirmed smaller fraction of dividing cells after 3D culture. Ki67 staining in AD-MSC neurospheres revealed that more than 80% of cells remains in quiescent phase [32]. Reduced proliferation of cells in spheroids suggested arrest in Go/G1 phase [7,8,26,33,34,35]. This observation could be connected with reduced CFU frequency indicating smaller number of clonogenic cells presented in 3D cultured population. However, many groups noticed better CFU frequency after transferring cells from spheres to 2D conditions [6,36]. Bartosh and colleagues published observations similar to ours, that CFU and population doubling values decreased directly after seeding cells derived from spheres. However, in further passages those features were similar or even higher than in a standard adherent culture [8]. Additionally, AD-MSC cultured as spheres expanded more rapidly than 2D cultured cells, but the difference was visible after 42 div of 2D culture [10]. Some research group did not observe the differences in CFU frequency between 2D and 3D cell culture [7].

Senescence was more pronounced in 3D cultured WJ-MSC. Increased β-galactoside activity was detected in almost a half of population just after changing the environment from 2D to 3D. To the contrast, other research groups reported that population after 3D culture contains less amount of senescent cells [6]. AD-MSC from spheroids contained more senescent cells in longer 3D culture, but still less than cells cultured on monolayer [10]. Differences in senescent processes between the results may arise from the source of MSC. In our experiments MSC were isolated from afterbirth tissue—part of umbilical cord. The initial population of WJ-MSC contained small number of senescent cells (less than 1%) and senescence process did not increase with further passages (up to 5% of senescent cells) [37], whereas for tissues from adult patients percentage of senescent cells was higher: for BM-MSC about 22,5% [6], for AD-MSC about 10% [10]. In our opinion, changing the culture conditions to less physiological for adherent cells (stress factor) accelerates senescence. Despite that, the other subpopulation that remains in the culture over time—those cells either have greater stress resistance or are developmentally younger and do not necessarily require adhesion for proper function.

These observations are consistent with changes in cell survival. In the majority of published experiments, 3D culture is a transient stage which last usually up to three div, no longer than seven div—especially when it comes to the use of low-attachment surface [4] among others because of poor MSC survival in 3D conditions. In our experiments 3D culture also reduced cell viability. Calcein AM and Ethidium Homodimer-1 staining pointed huge proportion of dead cells inside spheroid, which usually was placed in dark core. We noticed huge fraction of double stained cells, which was counted as dead—those cells were permeabilized with residual activity of esterases. Such situation was reported in other publication, using propidium iodide which has the same properties and emission spectrum as EthD-1 [38]. According to Peng, apoptosis rate in sphere reaches up to 20% of cells [39]. The most visible shift we noticed between third and fourth div of 3D culture. This change in viability was reported also by other research groups [8,32]. Even 3D cultures performed with other method, such as hanging drop, underwent the reduction in viability [8]. However, the spheroid culture contained more alive cells in 10 div of culture than in 3 div. With the duration of 3D cell culture, cellular composition of spheres had to change as the number of dead cells declined. This confirms our assumptions that during 3D culture, spheroid is decomposed, while population is segregated spontaneously—survived cells are more resistant to stress conditions, with slightly different properties than typical adult mesenchymal cells. Decreased viability might be also connected with size and insufficient transportation of nutrients and oxygen to all cells in bigger spheroids. For ESC embryoid bodies, oxygen concentration in the center can vary depends on the structure’s radius. In larger aggregates (400 µm radius) was lower than in smaller aggregates (200 µm radius) [40]. For 100 μm sphere diameter, the concentration of glucose and glutamine is 36,38 and 1,33 mmol/L—those values are insufficient, and cells begin to die in spheres core [41]. However, MSC spheroids are usually smaller. Hypoxic core, which could be responsible for increased cell deaths, was observed only in large spheroids consisting of 250,000 cells or more [27]. In smaller spheres oxygen gradient varied less than 10% across the aggregate layers. Decreased packing density might be the other mechanism that enable penetration of nutrients and oxygen to sphere core [27]. Observed population of smaller cells in 3D culture supported the evidence for change in cell densities and morphology in spheroids. Despite that, caspase activity confirmed that viability of cells decrease with sphere size [27]. Other used technique—thermal lifting in 3D culture was reported not only to lower apoptosis processes but also ischemic stress which is another relevant factor [36]. Cells in 3D culture were characterized by metabolism changes such as lower glucose consumption, lower L-lactase production [6,27], as well as changes in mitochondria [6]. Although lowered metabolism rate, there were observed higher level of mitochondrial and total reactive oxide species [6], what might be responsible for lowered viability and increased senescence during long-term 3D culture of MSC.

Except the changes of cell morphology, physiology and viability, 3D culture stimulates the acquisition of unique phenotype by cells. RNA-sequencing revealed the unique transcriptional profile of UC-MSC neurospheres, containing the features of NSC and MSC [42]. In our experiments, immunocytochemistry staining confirmed increased presence of early neural markers such as Nestin and β-III-Tubulin. 3D culture probably improve only the early stages of acquiring neural phenotype—NF-200 and NeuN expression was weak and did not changed between 2D and 3D culture. Yang and colleagues reported similar effect of 3D culture—cells from neurospheres expressed more Nestin after exposition to neural differentiation conditions, but any mature markers such as NeuN, MAP2, or glial marker GFAP [32]. However, 3D culture stage effected on future neural differentiation. Neurosphere culture of UC-MSC increased expression of neural [43] or both neural and glial [44] markers during neural differentiation. According to some reports, neurosphere culture of MSC improved neural differentiation potential [44]. Especially, Feng and colleagues claimed to have obtained even nerve-like cells with properties of astrocytes, neurons, and oligodendrocytes [45]. 3D culture could be an initial step preceding the neural differentiation under the specific differentiation conditions.

Stemness-related transcription factors (SRTF)—Sox2, Nanog, Rex1, Oct3/4, Klf4, are involved in cell divisions during embryonic development. Pluripotency and differentiation of MSC into all germ layers is broadly discussed. Oct3/4 was observed in WJ-MSC cultured in 5% oxygen concentration [46]. Nanog was detected in AD-MSC and BM-MSC, but not Sox-2 and Oct3/4 [47]. Although the discoveries of pluripotency genes in MSC, their expression is much weaker than in ESC [47] or iPSC [48]. 3D culture is proposed as a method to increase the expression of pluripotent genes by MSC. However, we did not observe the change of SRTF genes expression between 2D and 3D culture conditions what is not consistent with observations from other research groups. Pluripotent marker expression in MSC-neurospheres was observed by others in neurosphere-forming media [39,42,44] as well as in other sphere-inducing conditions [6,9,10,33]. Except the increased expression of SRTF, there were also observed epigenetic changes gene promotors and miRNAs responsible for pluripotency [9]. However, some research also did not report increased expression of stem cell markers in MSC-neurospheres—Bonilla-Porras and colleagues observed lower levels of Nanog, Oct4 and Sox2 in cells from WJ-MSC neurospheres than from 2D cultured WJ-MSC [43]. Dromard and colleagues did not notice the significant differences in expression of Nanog and Oct3/4 between spheres and monolayer [7]. The differences in above discussed results may be explained with the time of material collection. Cells in sphere did not exhibit pluripotency during whole period of 3D culture—expression was the highest between 3 and 5 div, after six div it drops [34]. We observed that even short time of adhesion change the RNA expression (Supplementary materials). Other authors reported that during 48 h after spheres return to 2D conditions, pluripotent expression decreases to the level presented in cultured monolayer cells [34]. Unfortunately, expression of pluripotent genes in MSC 3D still was significantly lower than in ESC [34].

It is discussed whether described effects are exaggerated by 3D conditions or could be influence by source of MSC. The comparative analysis are limited. WJ-MSC and BM-MSC generated secondary spheres after dissociation, while AD-MSC did not. What is more, secretomes profile of spheres were different—WJ-MSC secreted more neurotrophic factors. Protein level of neural markers inside sphere and under neural differentiation conditions also varied between different tissues. According to these observations, authors strongly suggested that WJ has better neurogenic potential than other sources [49]. In addition, Peng and colleagues performed identical analysis on MSC-spheres derived from two tissues—adipose and umbilical cord. The relative gene expression was similar, although the time of increase in gene expression differed. For some genes, such as Sox2 or Olig2 for UC-MSC spheres expression reached the maximum in second div, while for AD-MSC the peak was in seventh div [39,42]. It indicates that choice of MSC source be relevant.

Immunostaining revealed increased presence of surface marker SSEA4—glycosphingolipid which is included as a one of pluripotency markers. SSEA4 and SSEA3—other molecule from the same family—are characteristic for ESC cells [50]. SSEA3 is earlier in development—SSEA3 expression is the highest between four and eight cell stages of embryo, whereas SSEA4—morula and early blastocyst stages [51,52]. SSEA4 is also detected in early neuroepithelium [53,54] SSEA4-positive NSC can form neurospheres and SSEA4 expression remains sustained during 3 passages of NSC cultures [55]. Role of SSEA4 in NSC cells still remains unknown [51]. SSEA4 is also detected on MSC surface [18,56,57]. SSEA4 is presented during the MSC culture up to the ninth passage of WJ-MSC culture, while expression of SSEA3 is the highest directly after isolation and is detected only up to fifth passage [56]. SSEA4-positive BM-MSC population characterizes with better clonogenity [57]. SSEA4 together with small cell size are proposed as prognostic markers to distinguish young and old cells, which can be isolated with FACS [58]. SSEA4 expression may depend on cell culture conditions—it was correlated with medium serum concentration [56]. SSEA4, even counted as pluripotency marker, does not always coexist with other pluripotency genes what was shown on population of limbal stem cells (which resembled MSC) [59]. Similar situation was observed in MSC—SSEA4-positive WJ-MSC expressed SRTF genes on the same level as SSEA-4-negative cells [56]. Increased detection of SSEA4 in 3D cultured population might suggest acquiring this marker during spheroids culture or better survival of SSEA4-positive cells. Double staining for SSEA4 and Caspase-3 confirmed that apoptosis did occur only in a small fraction of SSEA-4-positive cells (Supplementary Materials). We rather assume that SSEA4-positve cells are more favorable to endure harsh conditions of neurosphere formation. The presence of SSEA4 positive cells could correspond with their undifferentiated state. According to Arrora and colleagues, undifferentiated state of cells in unfavorable conditions could be connected with quiescence [60]. Stress conditions such as loss of adhesion and high cell density lead to the quiescent state of MSC. Described earlier hypoxia and oxidative stress linked with sphere size [27] seems to be also responsible for cell persistent in a quiescent state in order to be available for further tissue repair and regeneration in beneficial [61].

In accordance with suggested neuroectodermal origin of distinct subpopulation of MSC [62], existing subpopulation of small cells and increased containment of early neural markers Nestin and β-III-Tubulin indicate that prolonged 3D culture enable to select cells with higher neural potential. Especially, very interesting for us was expression of Nestin, which may indicate the other explanation. Even though, quiescent NSC are negative for Nestin, active NSC express Nestin—importantly to mention, active NSC is the population which forms neurospheres, whereas quiescent NSC do not [63]. When it comes to MSC, Nestin may be the key explaining neurosphere formation—Nestin-negative cells do not form spheroids [64,65]. Nestin expression and sphere-forming capacities suggest neuroectodermal origin of this population [64]. Nestin-positive population derived from rat bone marrow contains MSC and Neural Crest Stem Cells as well [66]. During development, cells from neural crest which are not committed to glial lineage yet may migrate along nerves to bone marrow and there reside for the rest of life [67]. Those cells create a niche for hematopoietic stem cells (HSC) and remains quiescent in adulthood [64,65]. Under spheroids culture formed by magnetic levitation, increased Nestin expression is explained as quiescent state of such cells [67]. Neural crest-derived cells occur not only in bone marrow but also in other tissues containing MSC such as adipose [68,69]. Human umbilical cord blood contains neural crest-like progenitor cells which express Nestin, form neurospheres and can differentiate into neuronal and glial lineages [70]. Interestingly, we also reported more SSEA4-positive cells after long 3D cell culture. High content of SSEA3 and SSEA4 was observed during cell progeny activated by antibody mimicking Interferon-I [71]. Taking together, increased levels of Nestin and SSEA4 suggest quiescence of cells which remained over 20 div of 3D culture. Quiescent state can be the factor that enables survival of MSC during long-termed non-adherent conditions.

## 5. Conclusions

We confirmed that WJ-MSC can be cultured as spheroids on low-attachment surface with culture medium dedicated for neurospheres for at least 20 div. This type of culture characterizes with increased cell death after first three div, but then, viability stabilizes in later stage. Long-termed 3D culture of WJ-MSC as spheroids did not improved the cell condition. In fact, it reduced stemness and increased senescence process. However, it improved the occurrence of early neural markers what might indicate the survival of cell subpopulation with unique features, such as SSEA4 expression and possible quiescent state.

## Figures and Tables

**Table 1:** List of antibodies used for flow cytometry—Human mesenchymal stem/stromal cell (MSC) Analysis Kit (Beckton Dickinson) (cat. nr 562245).

	Antigen	Fluorochrome
Positive cocktail	CD73	APC
	CD90	FITC
	CD105	PerCP-Cy5.5
Negative cocktail	CD11b	PE
	CD19	
	CD34	
	CD49	
	HLA-DR	

**Table 2:** List of primary antibodies used for immunocytochemistry.

Antigen	Source	Isotype	Dilution	Company	Catalogue Number
Nestin	Mouse monoclonal	IgG1	1:500	Merck Millipore	MAB5326
β-III-Tubulin	Mouse monoclonal	IgG2B	1:500	Sigma-Aldrich	T8660
Neurofilament 200 (NF-200)	Mouse monoclonal	IgG1	1:400	Merck Millipore	N042
NeuN	Mouse monoclonal	IgG1	1:100	Merck Millipore	MAB377
A2B5	Mouse monoclonal	IgM	1:700	Merck Millipore	MAB312R
Ki67	Rabbit polyclonal	IgG(L+H)	1:1000	Abcam	AB15580
SSEA4	Mouse monoclonal	IgG3	1:400	Merck Millipore	MAB4304

**Table 3:** List of primers used for Real Time-Quantitative Polymerase Chain Reaction (RT-qPCR).

Gene	NCBI Reference Sequence	Product Size	Primer Sequence (5′ -> 3′)
β-Actin	NM_001101.5	250 bp	F: CATGTACGTTGCTATCCAGGC R: CTCCTTAATGTCACGCACGAT
Nestin1	NM_006617.2	64 bp	F: GGGAAGAGGTGATGGAACCA R: AAGCCCTGAACCCTCTTTGC
β-Tubulin III	NM_001197181.2	126 bp	F: GGAAGAGGGCGAGATGTACG R: GGGTTTAGACACTGCTGGCT
MAP-2	NM_001375545.1	99 bp	F: TTGGTGCCGAGTGAGAAGA R: GTCTGGCAGTGGTTGGTTAA
GFAP	NM_001363846.2	100 bp	F: CCGACAGCAGGTCCATGT R: GTTGCTGGACGCCATTG
Sox2	NM_003106.4	93 bp	F: GTGGAAACTTTTGTCGGAGA R: TTATAATCCGGGTGCTCCTT
Rex1	NM_001304358.2	107 bp	F: GCTCCCTTGAATGTTCTTTG R: GCCTGTCATGTACTCAGAAT
Nanog	NM_024865.4	103 bp	F: GAACCTCAGCTACAAACAGG R: CGTCACACCATTGCTATTCT
Oct3/4 (Pou5F1)	NM_001285986.2	331 bp	F: CCTGAAGCAGAAGAGGATCACC R: AAAGCGGCAGATGGTCGTTTGG

## Data Availability

The data presented in this study are available on request from the corresponding author.

## Supplementary Materials

The following are available online at https://www.mdpi.com/2073-4409/10/4/719/s1, Table S1: List of secondary antibodies used for immunocytochemistry, Figure S1: Secondary antibody staining controls for 2D and 3D conditions, Table S2: Population doubling time values (days) for 2D and 3D cultured WJ-MSC, Table S3: Quantitative data of immunocytochemistry staining analysis, Figure S2: Immunocytochemistry double staining for SSEA4 and Cleaved caspase-3 in WJ-MSC spheroid, Table S4: Relative gene expression level quantitative data. Figure S3: Extended analysis for relative gene expression level (fold change, mean ± SD) of neural and pluripotency phenotype in 2D and 3D WJ-MSC. Table S5: Relative gene expression level quantitative data for RNA collected directly from free floating spheroids.
