# Supplementary material for: Effect of Long-Term 3D Spheroid Culture on WJ-MSC
Source: Cells. 2021 Mar 24;10(4):719. doi: 10.3390/cells10040719 (PMC8063822; doi:10.3390/cells10040719)
Supplement: Supplementary file 1 [file cells-10-00719-s001.pdf]

cells

MDPI

Table S1. List of secondary antibodies used for immunocytochemistry.

Antibody	Conjugate	Dilution	Company	Catalogue number
Goat anti-mouse IgG1	Alexa Fluor 546	1:1000	Invitrogen	A21123
Goat anti-mouse IgG2B	Alexa Fluor 546	1:1000	Invitrogen	A21143
Goat anti-mouse IgGM	Alexa Fluor 546	1:1000	Invitrogen	A21045
Goat anti-mouse IgG3	Alexa Fluor 488	1:1000	Invitrogen	A21151
Goat anti-rabbit IgG (H+L)	Alexa Fluor 488	1:1000	Jakcson ImmunoResearch	111-545-144

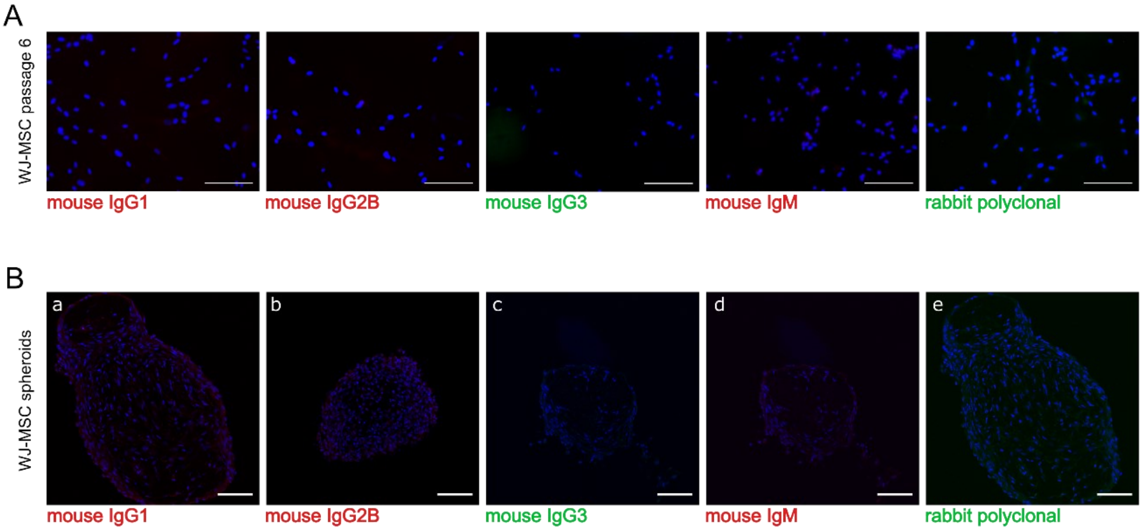

Figure S1. Secondary antibody staining controls for 2D (A) and 3D conditions (B). Following secondary antibodies were applies: goat anti-mouse IgG1 (a), goat anti-mouse IgG2B (b), Goat anti-mouse IgG3 (c), Goat anti-mouse IgM (d), Goat anti-rabbit IgG polyclonal (e). Following conjugates were applied: Alexa Fluor 488 – green label, Alexa Fluor 546 – red label. Antibodies were provided by Invitrogen, except goat anti-rabbit IgG (manufacturer: Jackosn ImmunoResearch). Cells used for control stainings: WJ-MSC from 6th passage (A) and WJ-MSC spheroids from 10th div (B). Scale bars: 100 µm.

Table S2. Population doubling time values (days) for 2D and 3D cultured WJ-MSC.

Passage n$_{o}$	Group	PDT values (days)	SD
3	Initial	1.29	0.08
7	2D culture	1.32	0.5
	3D culture	5.65	1.55
8	2D culture	1.6	0.3
	3D culture	1.94	0.88
9	2D culture	2.06	0.32
	3D culture	1.6	0.35
10	2D culture	2.06	1
	3D culture	1.78	0.03

Following groups of WJ-MSC were used: initial - 3rd passage WJ-MSC, 2D culture – 7th passage WJ-MSC, 3D culture – WJ-MSC cultured as spheres for 20 div. The results are presented as mean values of 3 experiments with standard deviation.

Table S3. Quantitative data of immunocytochemistry staining analysis.

Marker	Characteristic	Group	% of positive cells	SD
Nestin	Early neural	Initial	51.6	7.09
		2D culture	28.04	5.56
		3D culture	50.06	6.86
β-III-Tubulin	Early neural	Initial	65.55	1.66
		2D culture	42.28	2.84
		3D culture	85.66	7.9
NF-200	Neuronal	Initial	11	4.06
		2D culture	10.6	4.31
		3D culture	18.87	0.74
NeuN	Neuronal	Initial	28.88	3.5
		2D culture	16.56	2.66
		3D culture	15.7	5.95
A2B5	Glial	Initial	34.47	12.92
		2D culture	31.08	3.87
		3D culture	31.91	3.05
Ki67	Proliferative	Initial	50.55	5.48
		2D culture	20.31	2.39
		3D culture	7.88	2.05
SSEA4	Pluripotent/ embryonic	Initial	20.53	5.4
		2D culture	21.49	4.01
		3D culture	46.06	11.34

Following groups of WJ-MSC were used: initial - 3rd passage WJ-MSC, 2D culture – 7th passage WJ-MSC, 3D culture – WJ-MSC cultured as spheres for 20 div. The results are presented as mean values of 3 experiments with SD

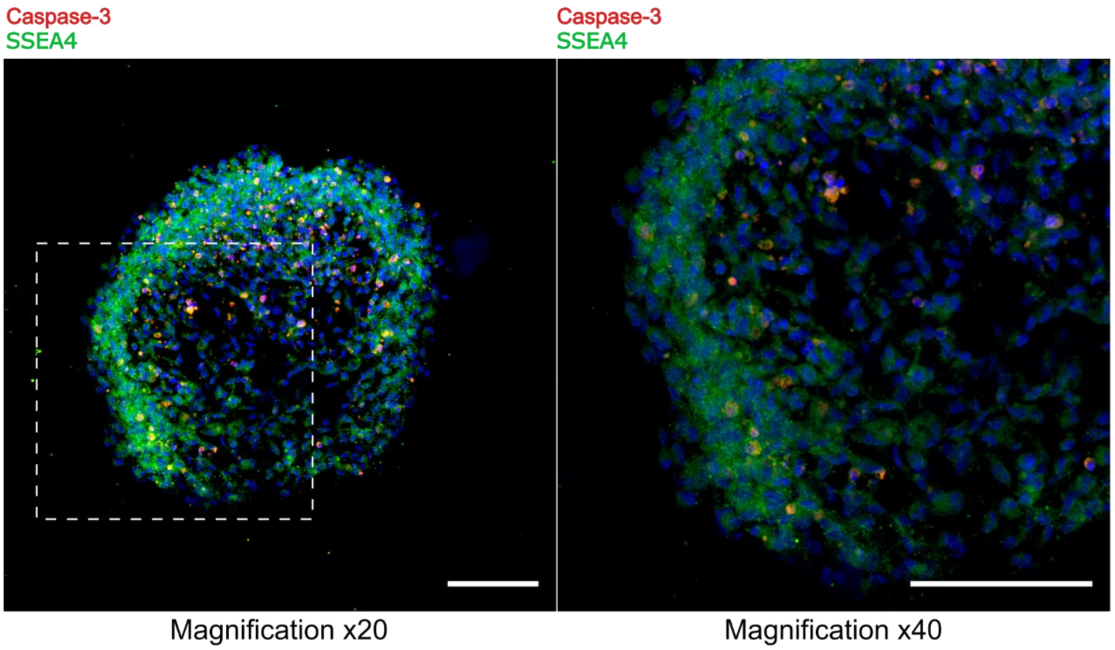

Figure S2. Immunocytochemistry double staining for SSEA4 and Cleaved caspase-3 in WJ-MSC spheroid. Population of SSEA-4 stem cells consists on both alive and dead cells, according to respectively positive and negative signals for Caspase-3, apoptotic marker. For Cleaved caspase-3 detection following antibodies were used: rabbit IgG polyclonal antibody (Cell Signaling Technology, cat. number 9661S) as primary antibody, Cy™3-conjugated goat-anti rabbit IgG polyclonal (Jackson ImmunoResearch, cat. number 111-165-144) as secondary antibody. Spheroid was collected in 10th div. Scale bars: 100 µm.

Table S4. Relative gene expression level quantitative data.

Gene	Characteristic	Group	RQ	SD
NESTIN	Early neural	Initial	1	0
		2D culture	0.6	0.16
		3D culture after 48 h	1.12	0.79
H3TUBULIN	Early neural	Initial	1	0
		2D culture	1.042	0.12
		3D culture after 48 h	0.37	0.28
MAP2	Neuronal	Initial	1	0
		2D culture	3.2	1.35
		3D culture after 48 h	1.05	0.32
GFAP	Glial	Initial	1	0
		2D culture	3.59	0.28
		3D culture after 48 h	0.6	0.38
NANOG	Pluripotent	Initial	1	0
		2D culture	1.69	0.48
		3D culture after 48 h	3.04	1.7
OCT3/4	Pluripotent	Initial	1	0
		2D culture	1.48	0.21
		3D culture after 48 h	1.07	0.7
SOX2	Pluripotent	Initial	1	0
		2D culture	1.63	1.46
		3D culture after 48 h	0.12	0.02
REX1	Pluripotent	Initial	1	0
		2D culture	0.09	0.07
		3D culture after 48 h	0.64	0.54

RQ – relative quantification of gene expression level. Following groups of WJ-MSC were used: **initial** - 3rd passage WJ-MSC, **2D culture** – 7th passage WJ-MSC, **3D culture spheroid** – WJ-MSC cultured as spheres for 20 div and then RNA was collected, **3D culture after 48 h** - WJ-MSC cultured as spheres for 20 div, transferred for 48 hours and then RNA was collected . For Rex1 3D spheroid group amplification after 40 PCR cycle was not observed in any experiment. Gene expression was normalized using β-actin (ACTB) and compared with the mean level of the corresponding gene expression in cells from initial population (3rd passage WJ-MSC). The results are presented as mean values of 3 experiments with standard deviation (SD).

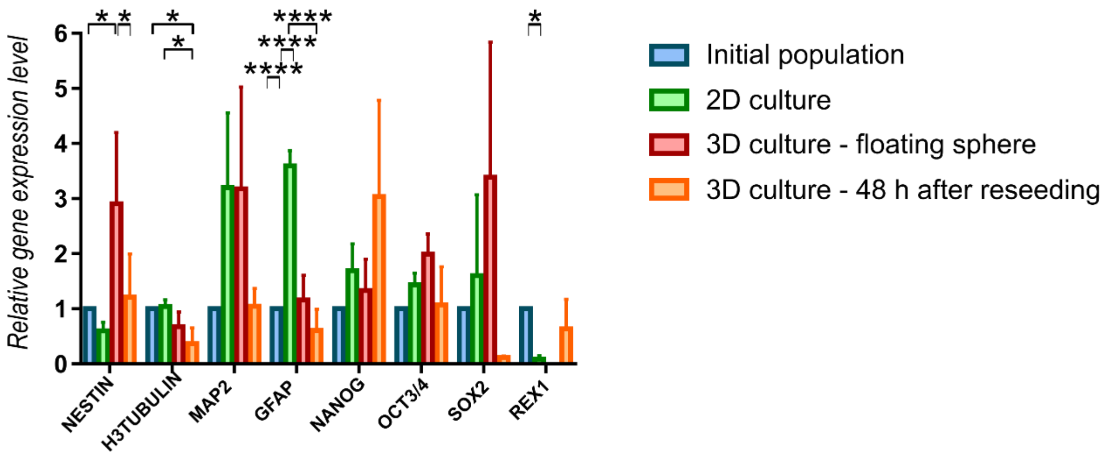

Figure S3. Extended analysis for relative gene expression level (fold change, mean ± SD) of neural and pluripotency phenotype in 2D and 3D WJ-MSC. Except initial population (passage 3rd) and 2D constantly 2D culture (passage 7th), we compared results for RNA collected directly from free floating spheroids and spheroid cultured for 48h in 2D conditions. Quantitation of these genes was determined relative to ACTB as a housekeeping gene by quantitative real-time PCR. Changes in gene expression in WJ-MSC cells cultured in 2D and 3D conditions are shown relative to that in cells grown in the initial population . Results shown are the mean of 3 independent RNA isolations, for * <0.05, ** <0.01,****<0.0001.

For 3D culture we wanted to examine differences between two timepoints of RNA collections – directly from floating spheroids and after more than 48 hours after transfer of spheroids to 2D culture conditions. We observed differences in results in both variants. We detected significant increase of Nestin expression in 3D spheroids – however, its expression decreased after return to 2D culture. This tendency was observed in other genes: for MAP2, Oct3/4 and Sox2. Interestingly, for Nanog situation is reversed: expression tended to increase in 48 h after reseeding spheroids to 2D conditions. To the contrary, level of Rex1 was for floating spheres were not determined – Ct value was greater than cut-off value for real-time amplification assays. Due to huge fluctuations between genetic material from different isolations we hardly reported any relevant statistical differences between all analysed groups in pluripotency genes. Change of expression pattern suggests that 3D culture effect might be transient or conditions modification drastically effect on cell characteristics. That effect was also observed by Pennock and colleagues, which measured pluripotent expression level in 5, 24 and 48 h after transferring spheres to 2D conditions (Pennock et al. 2015).

Table S5. Relative gene expression level quantitative data for RNA collected directly from free floating spheroids.

Gene	Characteristic	RQ	SD
NESTIN	Early neural	2.9	1.3
H3TUBULIN	Early neural	0.68	0.27
MAP2	Neuronal	3.18	1.8
GFAP	Glial	1.161	0.4451
NANOG	Pluripotent	1.33	0.57
OCT3/4	Pluripotent	1.993	0.37
SOX2	Pluripotent	3.387	2.45
REX1	Pluripotent	*undetermined*	-

RQ – relative quantification of gene expression level. For Rex1 3D spheroid group amplification after 40 PCR cycle was not observed in any experiment. Gene expression was normalized using β-actin (ACTB) and compared with the mean level of the corresponding gene expression in cells from initial population (3rd passage WJ-MSC). The results are presented as mean values of 3 experiments with standard deviation (SD).
